# Evaluation of Cytotoxicity and Genotoxicity of *Acacia aroma* Leaf Extracts

**DOI:** 10.1155/2014/380850

**Published:** 2014-10-28

**Authors:** C. M. Mattana, M. A. Cangiano, L. E. Alcaráz, A. Sosa, F. Escobar, C. Sabini, L. Sabini, A. L. Laciar

**Affiliations:** ^1^Área de Microbiología, Fac de Qca, Bqca y Fcia, UNSL, San Luis, Argentina; ^2^Área de Biología, Fac de Qca, Bqca y Fcia, UNSL, San Luis, Argentina; ^3^Área de Farmacognosia, Fac de Qca, Bqca y Fcia, UNSL, San Luis, Argentina; ^4^Departamento de Microbiología e Inmunología, Fac de Cs Ex. Fco-Qco y Nat., UNRC, Río Cuarto, Córdoba, Argentina

## Abstract

*Acacia aroma*, native plant from San Luis, Argentina, is commonly used as antiseptic and for healing of wounds. The present study was conducted to investigate the *in vitro* cytotoxicity and genotoxicity of hot aqueous extract (HAE) and ethanolic extract (EE) of *A. aroma*. The cytotoxic activity was assayed by neutral red uptake assay on Vero cell. Cell treatment with a range from 100 to 5000 *μ*g/mL of HAE and EE showed that 500 *μ*g/mL and 100 *μ*g/mL were the maximum noncytotoxic concentrations, respectively. The CC_50_ was 658 *μ*g/mL for EE and 1020 *μ*g/mL for HAE. The genotoxicity was tested by the single-cell gel electrophoresis comet assay. The results obtained in the evaluation of DNA cellular damage exposed to varied concentrations of the HAE showed no significant genotoxic effect at range of 1–20 mg/mL. The EE at 20 mg/mL showed moderate genotoxic effect related to the increase of the DNA percentage contained in tail of the comet; DNA was classified in category 2. At concentrations below 5 mg/mL, the results of cytotoxicity and genotoxicity of aqueous and ethanolic extracts of *Acacia aroma* guarantee the safety at cell and genomic level. However further studies are needed for longer periods including animal models to confirm the findings.

## 1. Introduction

For millennia, medicinal plants have been used in folk medicine. Simply, in recent times, scientific study of their effects has flourished. Nevertheless, some of them can cause adverse effects or have the potential to interact with other medications [1]. Moreover, there is little information on the potential risk to health of such herbs [2]. It is known that, in general, green plants are a primary source of antimutagens as well as natural toxic agents [3], and many plants contain cytotoxic and genotoxic substances resulting from the long-term use of such plants. In many places in Argentina, there is a rich tradition of using herbal medicine for the treatment of various infectious diseases, inflammations, and injuries [4–7]. Considering the vast potentiality of plants as sources for antimicrobial drugs, several authors have investigated the antimicrobial activity of medicinal plants [8–11].* Acacia aroma* Gill. ex Hook et Arn, whose common name is tusca, is a native plant of Argentina, widely distributed in central and northwest region [12]. This plant is used for wound healing and as antiseptic and for the treatment of gastrointestinal disorders. In Argentina, only studies on the antimicrobial activity of this plant in Tucuman and San Luis have been reported [13, 14] but there is little information about its toxicity. The cytotoxicity can be assessed by 2,3,5-triphenyltetrazolium chloride method (MTT), neutral red uptake, and others. The neutral red uptake assay (NRU) is a chemosensitive test that evaluates survival and cell viability, based on the ability of viable cells to incorporate the neutral red (NR), supravital dye. The genotoxicity can be assessed by* Allium cepa *test, comet assay, and others. Thus, the generation of DNA damage is considered to be an important initial event in carcinogenesis. In this study, our purpose was to contribute to the safe use of medicinal plants by means of the evaluation of the possible cytotoxic and/or genotoxic effects of* A. aroma* extracts by neutral red uptake and comet assays, respectively.

## 2. Materials and Methods 

Plant material aerial parts of* A. aroma* were collected in January–March of 2010, in the northwestern region of the province of San Luis, Argentina. Voucher specimens under the number 487 were deposited in the herbarium of the Botany Department, San Luis National University (UNSL). Leaves were used for the study.

### 2.1. Preparation of* Acacia aroma* Extracts Crude Ethanol Extracts (EE)

The* A. aroma *leaf powder was macerated in ethanol 95% (V/V) in a 1 : 3 proportion at room temperature, undergoing mechanical shaking for 4 h, followed by filtration. The extract obtained was concentrated in a rotavapor at 40°C. The vegetable residue was extracted twice again analogously, thereby obtaining the crude ethanol extract. Then, it was dissolved in dimethyl sulfoxide (DMSO) to achieve an initial concentration of 50 mg/mL, sterilized by filtration through a 0.2 *µ* membrane filter (Microclar), and stored at −20°C.

### 2.2. Preparation of Hot Aqueous Extract (HAE)

The* A. aroma* dried and powdered leaves (30 g) were macerated in water (1,400 mL) at 70°C for 120 min. This process was repeated twice. The extract obtained was filtered and lyophilized. To perform the assays* in vitro* the extract was solubilised in distilled water to achieve an initial concentration of 50 mg/mL and sterilized by filtration through a 0.2 *µ* membrane filter (Microclar) and stored at −20°C.

### 2.3. Cytotoxicity Assay

Cell culture cytotoxic assays were performed in Vero cells (*Cercopithecus aethiops* green monkey kidney epithelial cell line; ATCC CCL-81) grown in Eagle's minimal essential medium (EMEM) (Gibco, USA), supplemented with 10% (v/v) heat-inactivated fetal calf serum (FCS, Natocor, Argentina), glutamine (30 mg/mL), and gentamicin (50 mg/mL) (Sigma-Aldrich, Italy). Cell cultures were maintained at 37°C in a 5% (v/v) in CO_2_ humidified atmosphere.

### 2.4. Determination of 50% Cytotoxic Concentration (CC_50_)

Cell viability was determined by neutral red uptake test (NRU) [15]. Different concentrations of extracts were obtained by dissolution in Maintenance Medium (MM) (MEM + 2% FCS). They were tested in a range from 100 to 5000 *µ*g/mL of HAE and EE. Cell monolayers grown in 48-well culture plates (Cellstar, Greiner Bio-One, Germany) were incubated for 48 h at 37°C with different concentrations of extracts, in triplicate. Then, medium was removed and 500 *μ*L of NR solution (30 *μ*g/mL in MM) was added to each well. The plates were incubated once more for 3 h at 37°C to promote the uptake of the dye by cells. Subsequently, the supernatant was removed. The monolayers were washed with PBS, and 500 *μ*L of extraction solution (H_2_O : acetic acid : ethanol) (49 : 1 : 50) was incorporated in each well. After gently shaking the plates, the absorbance was read on a multiwell spectrophotometer (Bio-Tek, Elx 800) at 540 nm. Monolayers incubated only with MM were used as control. The CC_50_ was calculated from concentration-effect curves after nonlinear regression analysis (Boltzmann sigmoidal Origin 6.0). The results represent the mean ± standard error of the mean values of four different experiments.

Maximum noncytotoxic concentration (MNCC) was determined microscopically by daily observations of morphological cell changes for 72 h [16].

### 2.5. Genotoxicity Assay (The Comet Assay, Single-Cell Gel Electrophoresis)

Human blood was obtained by venous puncture from healthy, adult, young, and nonsmoking volunteers (with prior consent). Briefly, 50 *µ*L of heparinised whole blood was mixed with RPMI-1640, centrifuged at 1500 rpm for 5 min at room temperature, and incubated for 2 h at 37°C. Cellular viability was determined by exclusion method with Trypan Blue (0.4%). Fifty *µ*L of heparinised whole blood was incubated with HAE and EE at testing concentrations (1, 5, and 20 mg/mL) and incubated at 37°C for 2 h. Negative and positive controls were included. Comet assay was essentially performed as described by Singh et al. [17] with a few modifications: the cell suspensions were embedded in 100 *µ*L of 1% low melting point agarose (LMPA) and they were spread on a slide precoated with a film of 1% normal melting point agarose. Two slides were prepared for each sample in which agarose cell suspensions were allowed to solidify at 4°C. After the slides were transferred to lysis solution, pH 10, at 4°C for 1 h, slides were placed in an electrophoresis chamber exposed to alkali for 20 min by unwinding of DNA. Then, electrophoresis was performed for 20 min at 25 V/300 mA and electrophoresis slides were neutralized (three times) and stained with gel red (Biotium). The stained nuclei were visualized by fluorescent microscopic, photographed, and classified into four categories according to the average queue length (comet) ± standard deviation as follows: category 0 (no damage): 0 to 27 *µ*m; category 1 (low damage): 28 to 31 *µ*m; category 2 (medium damage): 32 to 35 *µ*m; and category 3 (high damage): greater than 36 *µ*m [18]. The rate of DNA damage for each sample was calculated using the following formula:

(1) $$\begin{array}{c} \text{DI }\left(\text{damage index}\right)=n_{1}+2n_{2}+3n_{3}+4n_{4}, \end{array}$$

where *n*_1_ are cells included in category 1, *n*_2_ in category 2, *n*_3_ in category 3, and *n*_4_ in greater damage. Bioassays were performed in duplicate and 200 cells were analyzed per treatment: negative control, positive control, and cells treated with plant extracts.

### 2.6. Statistical Analysis

The CC_50_ values were calculated from concentration-effect curves after nonlinear regression analysis based on Boltzmann sigmoidal curve by the software Graph Pad Prism 5.0. The results represent the mean ± standard error of the mean values of three different experiments. In all variants of Comet assay the values of descriptive statistics are shown as mean ± SD. The data were evaluated using nonparametric Jonckheere trend and Mann-Whitney tests. In all cases, the* a priori p* level for statistical significance was *α* = 0.05.

## 3. Results and Discussion

### 3.1. Cytotoxicity Assay

**Figure 1:**
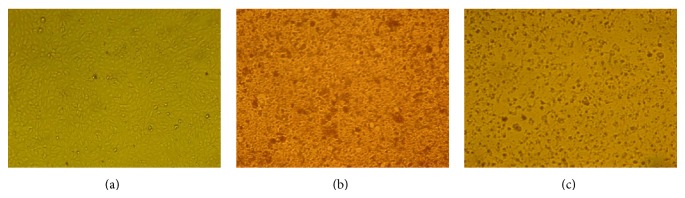
Morphological alterations of monolayers of Vero cells induced by* A. aroma* extracts, 20x. (a) Hot aqueous extract; (b) ethanolic extract; and (c) cell control.

**Figure 2:**
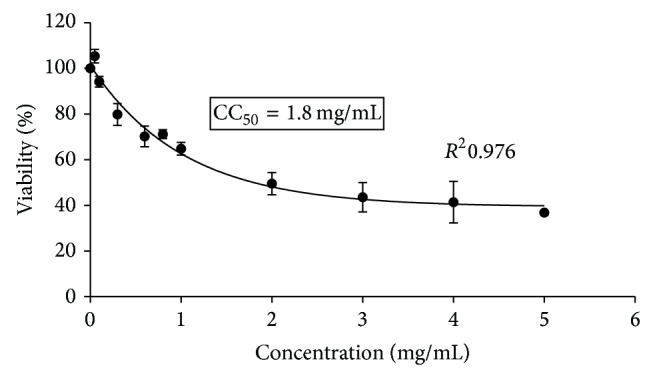
Percentage of viability of cultured Vero cells, incubated for 48 h in the presence of* A. aroma* hot aqueous extract at different concentrations determined by neutral red uptake (NRU). Each point represents the mean of four independent trials. CC_50_ was 1.8 mg/mL.

**Figure 3:**
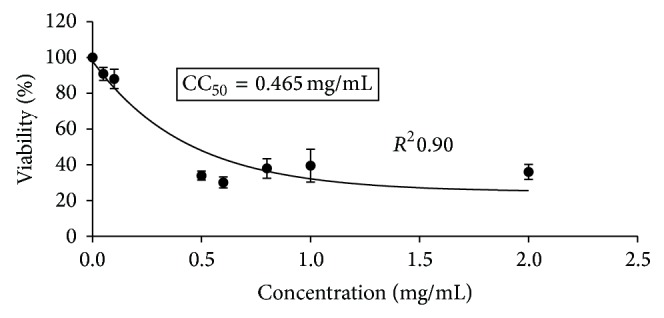
Percentage of viability of cultured Vero cells, incubated for 48 h in the presence of ethanolic extract (EE) of* Acacia aroma* employed at different concentrations determined by neutral red uptake (NRU). Each point represents the mean of four independent trials. CC_50_ was 0.465 mg/mL.

The neutral red is a weak cationic dye that readily penetrates cell membranes by nonionic diffusion and accumulates intracellularly in the lysosomes where it joins the lysosomal anionic matrix sites. Alterations in cell surface or membrane of the lysosome sensitive lead to lysosomal fragility and other changes that gradually become irreversible. Such changes caused by the action of xenobiotics result in a decreased uptake and binding of NR. Therefore, it is possible to distinguish dead, damaged, and living cells, which is the basis of this test. Cell treatment with a range from 100 to 5000 *µ*g/mL of HAE and EE showed that 500 *µ*g/mL and 100 *µ*g/mL were the maximum noncytotoxic concentrations, respectively (Table 1).  Figure 1 shows the morphological alterations of monolayers of Vero cells induced by cytotoxic concentrations of* A. aroma* hot aqueous extract (a) and ethanolic extract (b) with respect to cellular control that did not show any change (c). The cytotoxic concentration 50% (CC_50_) was tested by using neutral red uptake test. The results are graphically represented in Figures 2 and 3.  Figure 2 shows the percentage of viability of Vero cells, incubated for 48 h in the presence of* A. aroma* HAE at different concentrations. In this study, it was found that the CC_50_ value was 1.8 mg/mL for HAE. Previous studies in our laboratory [19] showed MIC values ranging from 625 to 1250 *µ*g/mL. On this base, for all microorganisms tested, this extract was not cytotoxic to Vero cells at bacteriostatics and bactericidal concentrations.  Figure 3 shows the percentage of viability of Vero cells, incubated for 48 h in the presence of* A. aroma* EE and the CC_50_ value was 0.465 mg/mL. At 48 h after treatment with the extracts and before the addition of NR, cell monolayers were observed under light microscope. It was possible to detect some structural changes in those cell monolayers treated with high concentrations of extracts with respect to cellular control that did not show any change (Figure 1). Monolayers treated with high concentrations of extracts exhibited holes formation with retraction of cells even attached and generated round cells grouped and refractile intracytoplasmic granulations, in addition to cell detachment. The CC_50_ value of EE was not cytotoxic to Vero cells at bacteriostatics concentrations (MIC: 78–156 *µ*g/mL) [19]. Our results are in agreement with those of Arias et al. [20]; they did not detect cytotoxicity in* A. aroma* extracts. Moreover, they have proposed this plant for pharmaceutical formulations.

Previous studies in our laboratory [14, 19] showed that the ethanolic extract of* A. aroma* had greater inhibitory power against* Listeria* and* Staphylococcus* compared to aqueous extract. This observation confirmed the evidence from a previous study which reported that alcohol is a better solvent for extraction of antimicrobial substances from medicinal plants than water [21] and, however, showed higher cytotoxicity in eukaryotic cells. The maximum noncytotoxic concentration was 0.1 mg/mL and the CC_50_ was 0.465 mg/mL, while for the HAE the maximum noncytotoxic concentration was 0.5 mg/mL and the CC_50_ was 1.8 mg/mL. Cytotoxicity similar values were obtained by the MTT method in our previous study [19]. In that study, the CC_50_ value was 658 *μ*g/mL for EE. For all microorganisms tested, this extract was not cytotoxic to Vero cells at bacteriostatics and bactericidal concentrations. 

### 3.2. Genotoxicity Assay

The single-cell gel electrophoresis (comet) assay is technically simple, relatively fast, and cheap, and DNA damage can be investigated in virtually all mammalian cell types without requirement for cell culture. The measurement of DNA damage can be used as a sensitive marker with great predictive value to detect the genotoxic properties of contaminant [22].

**Figure 4:**
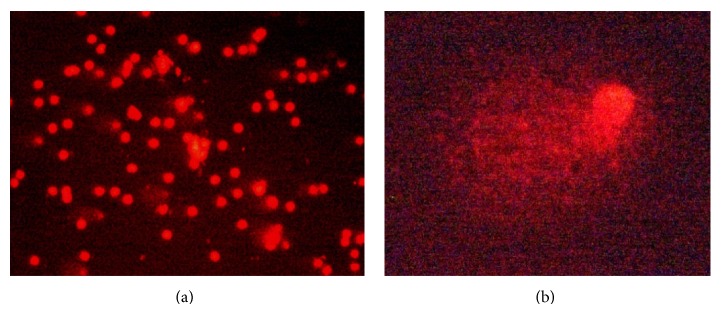
(a) Negative control (category 0). (b) Positive control, degraded nucleoids, and comet formation (category 3).

**Figure 5:**
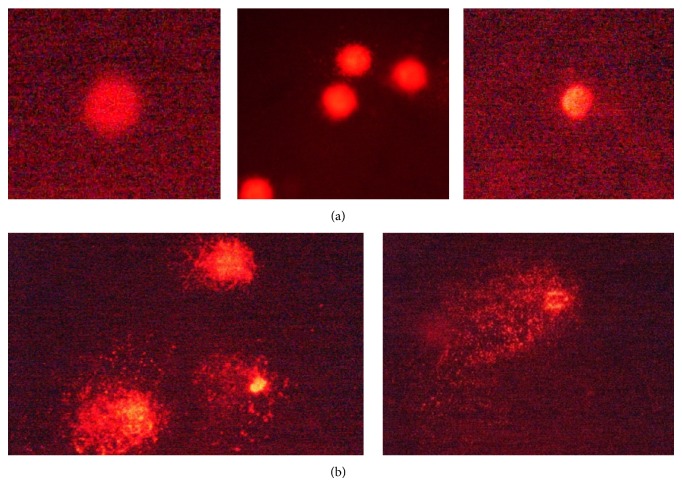
Evaluation of genotoxic effect induced by different extracts obtained from* A. aroma*. (a) Hot aqueous extract of* A. aroma *(1, 5, and 20 mg/mL): nucleoids without genotoxic damage (category 0). (b) Ethanolic and hot aqueous extracts of* A. aroma *at the highest concentration (20 mg/mL): nucleoids with mild damage (category 2).

**Figure 6:**
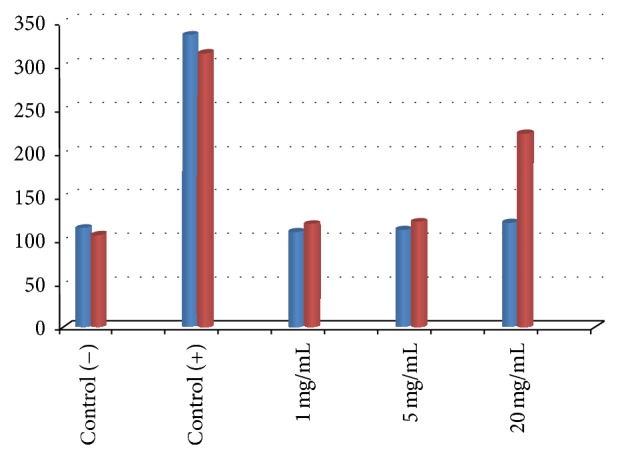
Damage index (DI) average of negative and positive controls and hot aqueous extract of* Acacia aroma*, at different concentrations (blue) and DI of negative and positive controls and ethanolic extract of* A. aroma*, at different concentrations (red).

The positive controls used in different experiments showed highly significant abnormal genetic changes: degraded nucleoids and comet formation (Figure 4(b)). The results obtained in the evaluation of DNA damage for effect of* A. aroma *HAE showed no significant genotoxic effect at concentrations ranging from 1 to 20 mg/mL (Figure 5(a)). The EE at 20 mg/mL showed moderate DNA damage, classified in category 2 (DI = 222) (Figures 5(b) and 6). DI values calculated for C (+) and C (−) were 106 and 314, respectively.

The results obtained in this work, under these experimental conditions, showed that, at all concentrations tested, the HAE of* A. aroma* was safe. It presents no cytotoxicity or genotoxicity, which is important considering that this is the part of the plant used as tizana by populations for the treatment of skin diseases and digestive ones. On the other hand, the EE at concentration of 20 mg/mL had moderate genotoxic effect. Varying levels of toxicity were found in other species of* Acacias*.   Cano Flores et al. [23] found genotoxicity levels above 1 mg/mL in* A. rigidula*. Arora et al. [24], by the comet assay of extracts of* A. nilotica*, detected statistically significant DNA damage only in the highest tested dose (2500 ppm). Studies of cytotoxicity and genotoxicity of* A. aroma* are poor or scarce. In Tucuman (Argentina), Arias et al. [20] evaluated the genotoxic activity of* A. aroma* by* Allium cepa* test and found dose-dependent effect. At concentrations of 1000 and 10000 ppm, they observed macroscopic and microscopic anomalies, which could be related to the properties of tissue regeneration and cicatrization of this plant, as well as its potential antitumoral activity. 

Sánchez et al. [25] considered that there are three different levels of DNA damage to be assessed by different methods. The first level is evaluated using assays that specifically detect damage breaks in DNA, the second level is produced by mutation in the genes, and the third level is evaluated by cytogenetic testing. In our study, the tests to rule out damage to the first level were performed. Other tests should be incorporated to further demonstrate the safety of both genetic level extracts of* A. aroma*. In summary, the results of cytotoxicity and genotoxicity of aqueous and ethanolic extracts of* A. aroma* guarantee, at concentrations below 5 mg/mL, the safety at cell and genomic level. Greater concentration of those extracts is necessary to inhibit bacterial growth (MIC up to 1250 *µ*g/mL). However, a literature survey also showed that plant extracts can be mutagenic as well as antimutagenic depending on the test system used. This indicates that a battery of assays is needed to reach a firm conclusion, for example, further studies for longer periods including animal models to confirm the findings.

## Figures and Tables

**Table 1:** Cytotoxicity of hot aqueous extract and ethanolic extract of *Acacia aroma* determined by neutral red uptake. CC_50_: cytotoxic concentration 50%.

*Acacia aroma* extracts	Maximum noncytotoxic concentration (MNCC) *µ*g/mL	CC_50_ by neutral red uptake *µ*g/mL
Hot aqueous extract	500	1800
Ethanolic extract	100	465
